# A work force model to support the adoption of best practice care in chronic diseases – a missing piece in clinical guideline implementation

**DOI:** 10.1186/1748-5908-3-35

**Published:** 2008-06-18

**Authors:** Leonie Segal, Kim Dalziel, Tom Bolton

**Affiliations:** 1 Health Economics and Policy Group, Division of Health Sciences, University of South Australia, GPO Box 2471, Adelaide, South Australia, 5001, Australia

## Abstract

The development and implementation of an evidence-based approach to health workforce planning is a necessary step to achieve access to best practice chronic disease management. In its absence, the widely reported failure in implementation of clinical best practice guidelines is almost certain to continue. This paper describes a demand model to estimate the community-based primary care health workforce consistent with the delivery of best practice chronic disease management and prevention. The model takes a geographic region as the planning frame and combines data about the health status of the regional population by disease category and stage, with best practice guidelines to estimate the clinical skill requirement or competencies for the region. The translation of the skill requirement into a service requirement can then be modelled, incorporating various assumptions about the occupation group to deliver nominated competencies. The service requirement, when compared with current service delivery, defines the gap or surplus in services. The results of the model could be used to inform service delivery as well as a workforce supply strategy.

## Background

The aging population and increasing rates of obesity mean that chronic diseases now represent a major health burden in most advanced societies, at an estimated 46% of global burden of disease and 59% of mortality [1]. Health is compromised when people with chronic conditions or risk factors are unable to access the mix of health services they need to prevent or manage their conditions. The development and publication of best practice guidelines for the management of chronic diseases has been used by clinical research groups and governments to promote the adoption of best practice care. This has resulted in the publication of evidence-based clinical guidelines for most chronic conditions (see Table 1), developed according to defined protocols, (as specified for instance in the National Health and Medical Research Council (NHNRC) of Australia Guide to the Development, Evaluation and Implementation of Clinical Practice Guidelines [2]).

**Table 1:** Proliferation of Clinical Practice Guidelines

Guidelines have been collected and displayed on internet websites including:
▪ Agency for Healthcare Research and Quality National Guideline Clearinghouse, USA; 2,097 guidelines, as at 27^th ^June 2007.
▪ NHMRC, Australia; 46 guidelines as at 29th June 2007.
▪ NZ Guidelines Group 2003; 73 guidelines and reports as at 27^th ^June 2007.
▪ National Institute for Health linical Excellence (NICE); 57 guidelines as at 27^th ^June 2007.
▪ The Guidelines International Network, which has a collection of 4,300 guidelines, systematic reviews, and evidence reports available to members (GIN, 29^th ^June 2007)

The adoption of care defined by clinical best practice guidelines is widely regarded as desirable, and the extent to which clinical practice conforms to best practice is one measure of health sector performance.

Despite the extensive publication and distribution of clinical best practice guidelines, there is ample evidence that large discrepancies between clinical care and best practice care persist and are associated with poorer health outcomes than achievable given the current state of knowledge [3-6]. We suggest that the observed departures from best practice care reflect a failure in one or more of the three conditions/enablers:

### 1. Sound knowledge of clinical practice guideline by clinicians

This requires that guidelines are written in a way that is clear to clinicians and translatable into actions and an effective dissemination strategy.

### 2. A practice environment supportive of delivery of best practice care

There are potential barriers at the practice level under the control of individual clinicians and practice teams, including factors such as practice culture, habit, motivation, attitudes, inadequate time or priority accorded to clinical best practice care, lack of equipment/infrastructure, or pertinent administrative processes.

### 3. A service system consistent with the delivery of best practice care

Important system level attributes, outside of the control of the clinician or practice, influence clinician and patient behaviour. These include financial incentives (payment arrangement for clinicians and user charges on consumers), quality audit/quality assurance and accountability arrangements, and a health workforce with the pertinent skills and competencies to deliver best practice care.

Most of the literature on implementation of clinical practice guidelines (CPGs) is focused on individual clinician or practice level approaches to changing clinician behaviour – the first two conditions above; [7-10]. Typical are the National Primary Care Collaboratives which seek to improve clinician's knowledge of CPGs but also support their implementation in primary care settings through culture change at the practice level [11]. Despite such initiatives, the quality of primary care does not conform to CPGs, particularly in the more disadvantaged communities [5,12,13].

It is postulated that without simultaneous attention to system issues, clinician and patient efforts to adopt best practice care will continue to falter. Examples of initiatives at the system level currently being pursued include the introduction of information technology (IT) systems into general practice that incorporate clinician decision support systems. This is likely to be most effective where combined with patient enrolment as we find in the UK and New Zealand [14]. Supportive funding models and quality assurance mechanisms are also critical. These are also receiving increasing attention [6,15,16]. What has received little attention to date is the workforce implication of best practice guidelines. Access to a suitably skilled workforce is a necessary condition to the delivery of and access to best practice care. The health workforce is a key system factor that must be in place to support the delivery of best practice care. Because of the large involvement of governments in the funding and delivery of health care, and the joint control over training and accreditation by governments and professional bodies, it is not a simple matter of assuming the 'market will respond' to supply the appropriate mix of skilled practitioners.

This means that the delivery of best practice care requires a complementary workforce strategy. In this paper, we describe a health workforce model designed to address this issue, and to estimate at the regional level the health workforce that would support the delivery of best practice care. The focus of the model is on the occupations and professional groups that are responsible for the delivery of competencies crucial to the prevention and management of chronic diseases in the primary care setting. This includes allied health disciplines, community nursing and medical, covering both current and emerging occupational groups.

Methods for estimating the desired level of health workforce are not well established. Little has been published on the economic 'market' for health competencies, especially in relation to allied health disciplines and especially on the demand side. Health workforce studies that exist typically focus on the supply of skilled health professionals (health workforce capacity), considering primarily issues of recruitment, training, retention, and career paths [17-19]. These include an examination of allied health services by Queensland Health, which focussed on factors that affect career satisfaction [20], or by Boyce [21], which focussed on allied health organisation structures, and a considerable program of work in the UK on health workforce planning for primary care [22,23]. This has been concerned in large part with increasing the capacity and efficiency of the health workforce. Strategies put in place to do this are have been broadly successful, although at considerable cost [23].

UK health workforce planning is also concerned with understanding workforce demand [24], largely at the primary care trust level and in the context of health services planning. Identifying future demand is a core component of the health workforce planning framework, but how this is to proceed is described in general rather than explicit terms [24].

Formal demand-side health workforce planning models go back to an early needs-based study of the medical workforce by Lee and Jones [25], a U.S. Department of Commerce planning process for the nursing workforce [26], and the Graduate Medical Education Advisory Committee GMENAC planning process for medical workforce in the US [27]. The GMENAC process represents the largest scale example of a needs model. It was designed to establish the future requirement for medical specialists, with the process involving consideration of the medical conditions managed by each specialty group, and the time commitment implied by agreed upon management protocols. The methodology was based upon a consensus approach, in which expert teams of clinicians agreed upon the typical/appropriate set of tasks and treatments to manage persons with conditions relevant to each specialty. This, combined with an assessed prevalence of particular conditions was used to estimate desirable levels of specialists per unit of population. The primary criticism of the study related to the assumption of 'fixed future technology'. The relationship between needs and service requirements was presumed to be static and did not account for the possibility of factor substitution. This is a valid concern and one that applies to many competing models, such as historic ratios, and needs to be accommodated.

The GMENAC model was designed to project 'need' for health professionals, not the demand which would be revealed in the medical market place. As noted elsewhere, because of market failure in the health care workforce, relying on expressed demand is unlikely to achieve an efficient or equitable solution. The distinction between 'demand' as defined by the numbers of health professionals needed to fill current positions, and the prior question of the number of positions required to meet community 'needs' is important. It is the latter concepts with which this paper is concerned.

Hurst has also taken the estimation of the health workforce market further, developing a comprehensive linked data set that could be used to explore both health workforce demand, as defined here, as well as supply. Thus far, the data set created has largely been used to provide comparative data, and exactly how it might be used to estimate demand is not yet described [28].

The wide-spread publication of best practice guidelines and progress in development of administrative data sets means a more objective basis for defining need and population health status is now possible. It is this question with which this paper is concerned. The Department of Human Services of South Australia implemented a quasi-evidence-based model in determining the allied health staff to deliver community-based diabetes care within the 'Hills Mallee Southern' Region [29]. The model relied on clinical and health services experts determining minimum skill requirements for the estimated population of the region with diabetes broadly based on best practice guidelines. This was translated into an effective full time equivalent EFT requirement and was used to negotiate staffing positions while taking into account budget constraints of the regional funder.

In general, approaches to health workforce planning (demand side) are highly simplistic. As noted by recent government-commissioned reports, health workforce models typically use either 'accepted' ratios (rules of thumb) of health workforce to population, 'expert opinion', or 'expressed demand' service use plus waiting lists [30,31]. While in a well-functioning market, expressed demand might represent a valid approach, it is flawed in relation to health [32], due to market failure that is precisely the reason why workforce planning is needed in the first place.

Given the failure to locate in the literature a sound evidence-based model for estimating the health care workforce, we have developed such a model. The logic of the model rests on the value to society of generating a health workforce capable of delivering best practice care. Non-acceptance of that presumption would represent a direct challenge to the entire clinical practice guidelines/best practice care movement. In short this paper describes a model to answer the question; 'What human resources are required to implement best practice CPGs in chronic disease management?'

#### The need-based community-based health workforce model

The focus of model development is on the sub-market of health professionals in their role in delivering community-based services in chronic disease management and prevention. This focus reflects the importance of multi-disciplinary team care in that setting, the extensive development of evidence-based clinical practice guidelines to support best practice care, and accumulating evidence that best practice care of chronic diseases for management and prevention is also cost-effective (*e.g*., [33-37]). Combined with the typically fragmented nature of service delivery, mixed public-private funding and incomplete knowledge by consumers of the effectiveness of health care, the skill mix will almost certainly be suboptimal in the absence of a health workforce planning.

The model describes a process for estimating the skill base required to deliver best practice care within a region, building on population health status and published best practice guidelines, and translated into a service requirement in the context of the local service system. The model is similar to a model developed in South Australia [29], but employing a more rigorous methodology and application. In implementing the model, it is expected it would in the first instance be applied to selected health conditions, covering all pertinent skill groups and competencies, but ultimately extended across all health conditions managed in the primary and community care setting.

**Figure 1:**
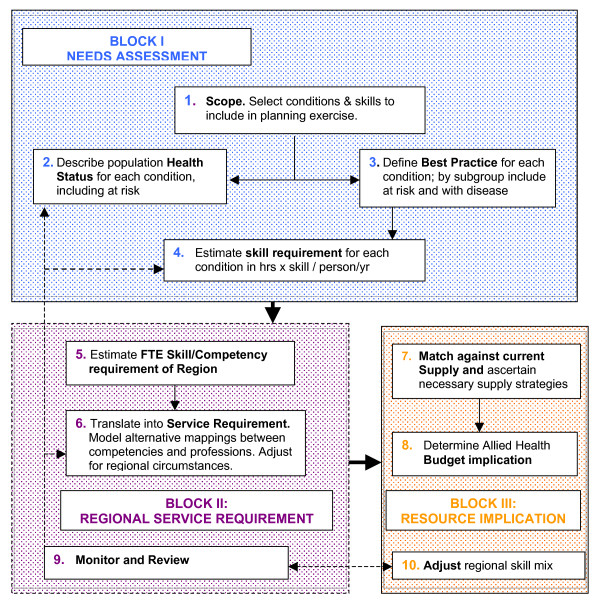
Allied Health Service Planning Framework and Tasks.

The model is illustrated in Figure 1 and described below. It includes a needs assessment task, as well as a process for translating skills into a regional service requirement and for assessing the strategic and budget implications. It also incorporates formal feedback mechanisms.

### I. Needs assessment

#### Task one: Scope

Scope involves selecting the target health conditions, skill groups, and geographic reach – national, state, regional, and/or local. The choice of condition could reflect the importance of the health problem within the region (number of persons affected, loss of quality of life/loss of life, costs of management) and the importance of multi-disciplinary care in treatment and prevention. Once the health condition(s) is selected, this suggests the pertinent skill groups and competencies that will need to be covered, which in turn suggests occupations to deliver the competencies. The principle is to scope occupations without regard to current regulatory and professional restrictions to reflect capacity to deliver nominated competencies. In translation to a health service model, the aim is to describe alternative scenarios which reflect alternative assumptions about relaxation of professional boundaries. The challenge in defining scope is to achieve a balance between the benefits of breadth of scope by including overlapping skills and approaches to management, and increasing complexity of the health workforce planning task.

#### Task two: Health status of the study region

This task involves estimation of the population with (and at risk of) target chronic conditions, and distinguishing subgroups by severity of condition and prevalence of specific comorbidities and pertinent socioeconomic variables. The aim is to define subgroups to match distinct management protocols while recognising constraints of administrative and other data sets.

#### Task three: Define best practice care

This task involves a systematic review of published CPGs for target conditions. The aim is to collate published protocols, distinguish subpopulations, including comorbidities, comment on quality of evidence, assess the level of agreement across guidelines, and address local applicability issues.

#### Task four: Skill requirement to deliver best practice care to each patient

This task involves interrogation of clinical protocols to describe distinct skills and competencies required to deliver best practice care for target condition(s). The aim is to estimate mean 'per patient' hours per year of care by distinct skill type or competency for each distinct patient subgroup. The effect of random and non-random variation, the latter capturing factors such as practice delivery models, should also be incorporated into the estimated skill requirement, while describing variation around 'best estimates'. It is expected that a clinical expert group would be established to assist in reaching consensus for this (and other) tasks, using standard methods such as Delphi, nominal group technique (NGT), or consensus development conference approaches [38].

### II Regional Services Requirement

#### Task five: Total skill requirements at the population level

This task involves translating the patient level estimates derived under task four into a regional skill requirement would involve relating those data to the population heath status estimates derived under task two. The result would be estimated total person hours/year by skill and competency required to support best practice care within the case study region, and adjusted to allow for non-patient related activities.

#### Task six: Regional workforce service requirement

This task involves translation of the estimated skill and competency requirement to a health workforce and identification of the feasible professional options for delivering each distinct skill, based on knowledge of associated competencies. The impact of using alternative professional combinations for delivering skills and competencies would be explored through modelling of plausible scenarios, such as altering the balance between specialist and generalist service providers, and consistency or not with current regulatory boundaries. Ideally, this task would be informed by evidence on effectiveness and cost-effectiveness of alternative professional delivery models (*e.g*., [39,40]). The result will be a number of plausible solutions that will exhibit differing levels of consistency with current professional boundaries, as defined by regulation, training, or professional practice.

### III Strategic implications for budget and workforce supply

#### Task seven: Workforce implications – matching demand against current supply

This task involves a *c*omparison between workforce demand, as estimated under task six with information about the current workforce supply. (The latter gathered from administrative data bases supplemented as necessary by or specific purpose surveys). The nature of any imbalance can be studied to identify nature of skill shortages (or areas of surplus). This can inform the work of planners in defining possible strategies to meet skill needs, in the short, medium, and long term, including implications for education and training.

#### Tasks eight: Budget or resource implications

The workforce estimate from task six can be translated into a regional workforce budget by applying standard wage rates and on-costs. Potential sources of funds and provision, and specifically the public and private mix, will need to be explored in the context of the health funding and delivery arrangements of the health system in question.

#### Tasks nine and ten: Monitor, review, and adjust

The model would need to be dynamic and respond to new clinical and service delivery information and changing regional characteristics. Ultimately model performance is measured by the extent to which care becomes more consistent with CPGs.

In Table 2, the model is further explained by describing it in the context of diabetes.

**Table 2:** Description of workforce model applied to type 2 diabetes.

**1 and 2. Scope and health status of the study region: **Diabetes selected as the target health condition. Establish health status (epidemiology) of regional population, reflecting an understanding of diabetes and protocols for prevention and management by interrogating available data sets; (for example as listed in Table 2). Describe number of persons with diabetes type (Type 2, Type 1, gestational) by disease stage – recently diagnosed, with specific comorbidities (vision impairment, neuropathy, foot problems, renal failure, heart disease), and persons at risk (*e.g*., combinations of IGT, obesity, previous gestational diabetes, high risk ethnic groups, aged over 50).
**3. Define best practice care: **Document clinical best practice for management of diabetes by type of diabetes and identifiable disease stages, highlighting the role of various skills. For persons with recently diagnosed NIDDM, describe optimal care over, say, five years in terms of consultations with diabetes nurse educator, podiatrist, dietitian, physical activity specialist; conduct a similar exercise for persons with specific complications and for persons at risk.
**4. Translate best practice protocols into skill requirement per person: **for the newly diagnosed diabetic, persons with specific comorbidities and complications, and persons at risk. Express as mean hours by allied health skill/person/year at each disease stage, *i.e*., hours/persons for S_a1 _to S_ai......... _S_n1 _to S_ni _W here: S_ai _is skill type 'a' (*e.g*., dietetics) for population subgroup 'i' (*e.g*., person with newly diagnosed NIDDM).
**5. Translate mean hours into an EFT skill requirement for each skill type: **(podiatrist, dietitian, diabetes nurse educator, etc.), by combining mean hours for each skill type per person per year with estimated numbers in each diagnostic category
Multiply (S_a1 _to_... _S_n1_) × H_1........ _to (S_ai. _to S_ni_) × H_i_.
Where Hi is number of persons in disease category/stage
Adjust for typical contact hours per occupational group to arrive at EFT requirement. Consider whether aim is to achieve best practice care or 'acceptable' care, and what this might mean.
**6 and 7. Translate skill requirement into a service requirement and match against current supply: **by taking results from step 5 together with local knowledge of allied health workforce, opportunity for multi-skilling or specialised care, geography of region, distribution of population, possible approaches to program delivery, nature of the client population. Compare with current skill mix and service structure.
**8. Establish budget implications: **Determine funding level required to support the projected service requirement. Compare with current resourcing levels. Consider how funding might be split between levels of government and program area. Consider balance between private and public funding.
**9 & 10. Monitor, review and adjust: **Create a plan for frequency of revision and adjustment based on nature of evidence for diabetes and characteristics of the region.

### Implementation issues: Data

Despite the pace of construction of clinical guidelines there are still gaps in the available evidence. This will impinge on the capacity to implement the workforce planning framework across all health conditions, given the reliance on published CPGs. On the other hand, increasingly, standard data collections can be interrogated to meet other information requirements of the model. An example of pertinent data sources for Australia that can be used to determine population health status are listed in Table 3. The ability to implement the model in a way that is truly evidence-based cannot be established in principle, but only in the context of a specific application.

**Table 3:** Example of Australian Data Sources that might be used to establish health status of regional population

**Routine National Surveillance Data**	
Census data	Age, gender, socioeconomic index, ethnicity, etc.
Morbidity and Mortality	National Death Index, Burden of Disease Studies[44]
Regular surveys	National Health Survey, ABS Cause of Death statistics etc.
**Administrative data sets**	
Hospital data bases	Inpatient minimum datasets, Outpatient minimum datasets
Medicare data	Medical services MBS (Medical Benefits Schedule) on-line data,
	Prescription pharmaceuticals PBS (pharmaceutical benefits schedule) online
Specialist insurers	Veterans Affairs, Transport Accident, WorkCover, etc.
**Disease/condition specific, cohort data**	
Disease Registers	Diabetes, Cancer, joint replacement register
Special Surveys, including	Screening surveys; Region-specific (*e.g*., Busselton, Dubbo cohort studies), Record-linkage studies, etc.
Primary care data sets	Divisions of general practice; Primary care collaboratives

Note: Full references to all data sources available through correspondence with authors

## Discussion

There are important conceptual and technical challenges of model implementation that are discussed here.

### Summation of service needs across conditions

Because of the possible overlap between conditions and management, given common co morbidities, it is preferable that all chronic conditions are included in a single workforce planning exercise. Regardless of scope of the exercise, it will be important to adjust for the fact that some services will address more than one disease/health condition.

The existence of comorbidities is not only pertinent in terms of possible synergies in components of management, it may also influence approaches to management. For example, a high proportion of persons with Type 2 diabetes, also have Coronary Heart Disease (CHD), CHD risk factors, or serious mental health problems (*e.g*., [41,42]). Psychiatric co-morbidities not only represent a health problem to be managed, but they may impact on the ability of individuals to comply with recommended care (for both the psychiatric condition and their other comorbidities). This suggests the need for alternative, more intensive approaches to management [43].

### Diagnostic criteria: 'The Clinical Iceberg'

There is considerable scope for imprecision in estimating the numbers of people with particular conditions. Typically chronic diseases (such as CHD, hypertension, and Type 2 diabetes) as well as risk factors occur across a range of severities. As the disease or risk factor becomes milder, the frequency becomes greater. As described in the model, in estimating the population health status, numbers will need to be estimated for various sub-populations – such as those at risk, those with single conditions and those with comorbidities – and categorised by disease stage and severity. Ideally, subgroups should be defined, wherever there are differences in the optimal approach to management and prevention. The problem is that the number of people identified with a condition depends not just on population characteristics but also on diagnostic criteria, which are necessarily somewhat arbitrary. Thus the distinction between the general population, persons at risk, and those with established disease can be indeterminate, changing with the understanding of the disease and with known interventions for prevention, ameliorating symptoms, or modifying disease progression. For example, the level of blood cholesterol predicts the risk of ischaemic heart disease mortality, rising across the entire range of cholesterol levels in the population, and therapeutic reduction in those levels by diet and/or drugs reduces the risk. Thus defining a population with high cholesterol is somewhat arbitrary.

Application of the model will also depend on the nature of available data sets from which subpopulation estimates will be derived.

### Interpreting CPGs

#### Variability in client needs

In interpreting clinical practice guidelines and translating these into a skill and competency input required for individual management, the varying needs of client subgroups must be considered. This covers not just those with distinct comorbidities as discussed above, but also persons from specific cultural or socioeconomic groups. Variable time inputs required for the effective management of clients with differential risk and different capacities to respond to care should be allowed for. There is potential for this to be ignored if modelling is unduly focused on the typical client.

#### Technology of care delivery

Approaches to care delivery change over time with new understandings about disease processes and impact of care, access to new treatments, and the influence of cost pressures, etc. In expressing requirements in terms of competencies rather than occupations, this will more readily allow modelling to consider likely factor substitution (between health professional groups), and the possible substitution between the formal and informal care sector. However, where technology change means a shift in competencies that are recognised in revised guidelines, this can only be accommodated by adjusting the model periodically to reflect new information, which should be built into the planning cycle. Attempting to predict new technology is unlikely to be successful.

#### Relationship between service delivery and access to care

Another issue is whether the health workforce model, even if successfully implemented, will impact as hoped on quality of care. While service levels derived from the model are designed to ensure that all persons with nominated conditions are able to access best practice care, this does not ensure demand by patients reflects their level of need. This will depend on service characteristics, such as accessibility, perceived quality, cultural relevance and cost to the user, and patient characteristics. Thus, even if such a workforce planning model were implemented and translated into services, there could still remain a mismatch between demand and need. Promoting an understanding of best practice guidelines and promoting appropriate use of services would still be required. This understanding would not just be about staffing and services, but also about appropriate settings.

Furthermore, the strength of the underlying evidence base will vary between guidelines, as will the incorporation of considerations of cost effectiveness. Both factors will impact on patient outcomes even if guidelines are successfully implemented as intended.

As noted in the description of the model, how the skill requirement is translated into a service model and staffing requirement will depend, in part, on views about the relative role of specialist and mainstream/generic service delivery providers. This decision will be informed by matters such as published evidence, the service philosophy, the size of the region, capacity to attract specialist and generalist staff, the mix of conditions included in the health services planning exercise, views about critical mass and professional development, adequacy of the training of health professionals, and capacity to allocate time between competing pressures.

One of the strengths of the proposed model is that it provides an opportunity and framework in which to analyse the impact of varying assumptions about definition of disease and at-risk populations, estimating skill inputs from care protocols, and translating skills and competencies into professional groupings or occupations. It is proposed that an expert clinical and policy advisory committee would be established as part of model implementation to inform the sets of assumptions incorporated into the modelling.

The performance of the model is to be assessed in the first instance in terms of capacity for implementation; this essentially concerns access to necessary data and ability to develop robust sets of assumptions to complete the analysis. The ultimate test of performance must also include whether it is found to offer a useful contribution to workforce planning, health services planning, education, and training policy, and whether these in turn support the adoption of clinical best practice care and are expected improvements in patient health outcomes.

## Conclusion

While undoubtedly there are important practical and theoretical issues still to be explored, as enunciated above, we suggest that adopting a health workforce demand model similar to that described here is critical to moves towards best practice care. Unless the service system has the skill-base to deliver best practice care, the value of guideline development and dissemination will be compromised in its capacity to deliver best practice care. Given distortions in the market for health care and restrictions on health workforce supply, it is highly unlikely that the normal market mechanisms will resolve the health labour force question in a way that will support delivery of best practice care.

The proposed health workforce model is an important first step in developing an evidence-based human resources framework for implementing chronic disease management consistent with clinical practice guidelines that offers an evidence-based alternative to the commonly used simplistic methods (like population ratios). Application of the model will allow planners to determine the gap between the current health workforce and that required for evidence-based practice to inform service planning as well as education and training policy.

The achievement of best practice care and enhanced health and wellbeing of persons with chronic disease presumes however that related health system reform elements are simultaneously pursued. It is also the case that if clinical guidelines are not based on evidence regarding effective and cost-effective care, then supporting the delivery of care consistent with clinical guidelines will not achieve the promised gains in health and wellbeing. For this reason, the ultimate test of model performance is whether clinical practice is better aligned with CPG, and whether through this the expected improvement in patient outcomes are realised.

While there are undoubted challenges in the implementation of the proposed model, it provides an evidence-based alternative to the commonly used simplistic methods. Subsequent application will provide information about the gap between the current skill base and that required for evidence-based practice, highlighting priorities for change to inform health care reform, education, and training policy. The ultimate test of the model is whether its use results in changes in clinical practice in alignment with CPG-defined care.

## Competing interests

The authors declare that they have no competing interests.

## Authors' contributions

LS designed the model, conceived its application to chronic disease and drafted the manuscript, KD contributed to model design and helped to draft the manuscript, TB assisted with research and drafting of the manuscript. All authors read and approved the final manuscript.
